# Rheumatoid Lung Disease

**DOI:** 10.31138/mjr.050325.erl

**Published:** 2025-09-30

**Authors:** Alexandros A. Drosos, Anastasia K. Zikou, Melina Yerolatsite, Paraskevi V. Voulgari

**Affiliations:** 1 Department of Rheumatology, School of Health Sciences, Faculty of Medicine, University of Ioannina, Ioannina, Greece;; 2 Department of Radiology, Medical School, University of Ioannina, Ioannina, Greece;; 3 Department of Medical Oncology, University Hospital of Ioannina, Ioannina, Greece

**Keywords:** rheumatoid arthritis, lung manifestation, ILD, screening, HRCT, RA-pathophysiology

## Abstract

Rheumatoid arthritis (RA) is a chronic systemic inflammatory disease that affects the synovial membrane of the joints in a symmetrical pattern, leading to cartilage and bone damage. It affects 0.5–1% of the general population, predominantly women in their fourth to fifth decades of life. In addition to its impact on the joints, RA has numerous extra-articular manifestations involving the skin, eyes, heart and lung. Among these, pulmonary manifestations play a pivotal role in morbidity and mortality. RA can affect all anatomical compartments of the lung such as the airways, lung parenchyma, pulmonary vasculature and pleura. Among these, parenchymal disease that manifests as interstitial ling disease (ILD) is the most serious. The etiopathogenesis of RA remains elusive with multiple underlying genetic and environmental risk factors implicated. Cigarette smoking has been strongly associated with an increased risk of developing RA in genetically susceptible individuals, particularly those carrying the shared epitope of the human leucocyte antigen (HLA) DRB1* alleles. Smoking induces chronic pulmonary inflammation, promotes protein citrullination in the lungs, leads to the generation of anti-citrullinated antibodies and contributes to the development of RA. In this review we discuss the various manifestations of RA associated lung disease, methods of screening, the pathophysiology of RA in relation to the lung and recent advances in treatment.

## INTRODUCTION

Rheumatoid arthritis (RA) is a chronic inflammatory disease that predominantly affects females in the fourth to fifth decades of life. It impacts 0.5–1% of the general population and involves primarily the peripheral joints, in a symmetrical pattern, causing synovitis, which leads to joint damage and bone destruction.^1^ Beyond its impact on the joints, RA is associated with several extra-articular manifestations (EAM), affecting the skin, eyes, heart and lung, that are responsible for a significant rate of morbidity and mortality.^2,3^ The etiology of RA is multifactorial, involving an interaction between the genetic predisposition, such as the presence of the ΄shared epitope΄ (SE) and environmental factors.^4^ Among these, infections, cigarette smoking, air pollution and other environmental exposures may play an important role in RA pathogenesis. RA is classified as seropositive in the majority of cases (70–80%), with patients exhibiting autoantibodies in their serum including rheumatoid factor (RF) and/or autoantibodies to citrullinated protein antigens (ACPA). In contrast, 20–30% of patients lack these autoantibodies and are classified as seronegative.^5,6^ The clinical diagnosis of RA requires the presence of persistent inflammatory arthritis lasting at least 6 weeks, according to either the 1987 American College of Rheumatology (ACR) revised criteria,^7^ or the 2010 ACR/European Alliance of Associations for Rheumatology(EULAR) classification criteria.^8^

Circulating RA autoantibodies can be detected three to five years or even longer, before the onset of clinical arthritis. This phase of autoimmunity, occurring in the absence of joint disease is referred to as “preclinical RA” and suggests that the disease may originate at an anatomical site outside the joints.^9,10^ Among the EAM of RA, lung involvement is common, often emerging within the first five years of disease onset. While musculoskeletal symptoms typically precede the respiratory manifestations, in approximately 20% of patients, pulmonary symptoms are the initial presentation,^11,12^ supporting the hypothesis that the lungs may be a primary site for the initiation of RA-related autoimmunity. In this review, we present the various manifestations of RA associated lung disease, its diagnosis and treatment and the potential role of the lungs in RA pathogenesis. For this reason, we conducted a comprehensive literature search through PubMed/Medline, Scopus and DOAJ until March 2025, using the following search terms: “RA and lung disease”, “RA and ILD”, “RA and lung involvement”, “RA and lung manifestations”, “RA and pleurisy”, “RA and airway disease”, “RA lung disease and treatment”, “RA-ILD and treatment”, “RA lung disease and RA pathophysiology”.

## LUNG INVOLVEMENT IN CLINICALLY CLASSIFIABLE RA

Pulmonary manifestations are common in RA and are directly associated with the RA disease process itself, or are related to pulmonary infections or drug toxicity. RA affects all the anatomical compartments of the lung, including the airways, parenchyma, pulmonary vasculature and pleura. Each of these lung regions exhibits distinct anatomical, clinical, functional and imaging characteristics, as summarised in **Table 1**. Airway disease involves inflammation of both small and large airways and is typically identified on high-resolution computed tomography (HRCT) scan as bronchial wall thickening, centrilobular opacities consistent with bronchiolitis, abnormal air trapping and bronchiectasis. Pulmonary function tests (PFT) usually demonstrate obstructive pattern characterised by decreased forced expiratory airflow, with prevalence ranging from 8–26%. Parenchymal disease appears on HRCT as nodular, ground glass opacities, alveolar infiltrates, honeycombing changes and fibrotic reticulation. These parenchymal changes are collectively referred to as interstitial lung disease (ILD), with reported prevalence ranging from 7 to 79%.^13–16^ PFT typically reveal a restrictive pattern and reduced diffusion capacity. Pleural disease may occur as an initial clinical manifestation of RA and can be identified on imaging in approximately 50% of cases.^13–16^ There is significant variability in the reported prevalence rates of lung involvement in RA, due to several factors such as the heterogeneity of patient populations studied. Early RA, established disease, seropositivity, and smoking may influence the prevalence of lung disease. In addition, there is a variability in the description of lung disease, due to different diagnostic modalities used for lung assessment such as chest X-rays, HRCT, PFT and lung ultrasonography. Most studies demonstrate that HRCT is the most sensitive tool for detecting lung involvement in RA patients. Furthermore, the timing of lung assessment appears to influence both the frequency and the type of lung involvement. Several studies have demonstrated a high prevalence of airway disease in early RA. Wilsher et al. used HRCT in patients with early RA and found airway disease to be more prevalent than parenchymal lung disease (82% vs 23%).^17^ In a controlled study, using HRCT in early RA, we also identified high prevalence of airway disease (air trapping in 69% and bronchiectasis in 58%), while parenchymal disease, such as ground-glass opacities, was observed less frequently (35%).^18^ However, other studies on early RA have reported a higher prevalence of parenchymal lung disease compared to airway disease.^13–16^

**Table 1.** Lung involvement in rheumatoid arthritis.

	**Airways**	**Lung parenchyma**	**Lung pleura**	**Lung vasculature**
**Anatomy**	Bronchi Bronchioles Terminal bronchioles	Alveoli Interstitium between lung and lung capillaries	A thin membranous lining surrounding the lung	Pulmonary arteries and veins Bronchial arteries and capillaries
**Clinical disease manifestations**	Asthma Bronchitis Emphysema	Interstitial lung disease	Pleurisy Pleural effusion	Pulmonary artery hypertension Lung haemorrhage
**Imaging findings**	Air trapping Bronchial wall thickening Bronchiectasis Centrilobular opacities	Nodules Ground glass opacities Reticulation Honeycombing	Pleural thickening Pleural effusions	Mosaic attenuation Pulmonary artery enlargement
**Pulmonary functions tests**	Obstruction Elevated residual volume	Restriction Decreased diffusion capacity	Reduced lung volume	Decreased diffusion capacity

## RISK FACTORS FOR LUNG INVOLVEMENT IN RA

Several risk factors for lung disease in RA have been reported, including longstanding disease, older age, disease severity, male sex, tobacco exposure, exposure to air pollutants and dust, as well as the presence of autoantibodies. Some studies suggest that different risk factors may be associated with different types of lung involvement. Specifically, ACPA autoantibodies have been primarily associated with airways disease, while RF positivity has been associated with parenchymal disease.^13–19^ However, other reports found the opposite results.

The association between the number and type of different ACPA specificities and lung abnormalities in early untreated RA was investigated by Joshua et al. They found that specific ACPA and RF isotypes were associated with the presence of parenchymal lung disease and airway abnormalities at disease onset, prior to the initiation of any treatment of RA.^20^ While autoantibodies play a significant role in RA-associated lung manifestations, novel lung disease specific biomarkers have also been identified. Gochnico et al. found higher levels of interferon-γ (IFN-γ) and transforming growth factor-beta (TGF-β) in bronco- alveolar lavage (BAL) fluid in RA patients with ILD.^21^ Other investigators reported an association between specific ACPA targeting citrullinated HSP90 and RA-ILD.^22^ Recently, Aripova et al. reported that serum antibodies against malondialdehyde-acetaldehyde (MMA) albumin, MMA collagen, MMA fibrinogen, MMA vimentin were associated with the development of RA-ILD.^23^ A gain-of-function variant r535705950 in the promoter of MUC5B, which encodes mucin5B, has been linked to idiopathic fibrosis.^24^ Recent studies have demonstrated that this MUC5B promoter variant is also associated with RA-ILD, with evidence of UIP pattern.^25^ However, all of these bio-markers considered require further validation in larger, well designed studies before they can be routinely applied in clinical care.^26^

In a retrospective study, Dinache et al. assessed lung disease in RA and found that ILD developed within the 5 years from RA diagnosis. Furthermore, image findings associated with ILD, such as chest X-rays and HRCT were more prevalent among men, seropositive patients and those receiving methotrexate (MTX).^14^ In contrast, Kim et al. reported that older age at disease onset, high body mass index (BMI), smoking and oral steroids were associated with RA-ILD while, MTX use was less likely to be associated with ILD.^27^ MTX is the key anchor drug in the treatment of RA. However, whether MTX exposure increases the risk of ILD in RA patients is disputed. In a multi-ethnic case control study, Juge et al. examined the association between MTX use and ILD development in RA patients. The study included 410 RA-ILD patients who had been exposed to MTX and 673 RA patients treated with MTX but without ILD. The results showed that MTX use was not associated with an increased risk of developing RA-ILD.^28^

Similarly, another study investigated the relationship between MTX treatment and lung disease risk in a nationwide population-based RA cohort study. Data were retrieved from the Danish National Patients Register and the DANBIO registry for rheumatic diseases including 30512 RA patients registered between 1997 and 2015. The authors concluded that MTX use was not associated with an increased risk of ILD or respiratory failure in RA patients.^29^

**Figure 1.**
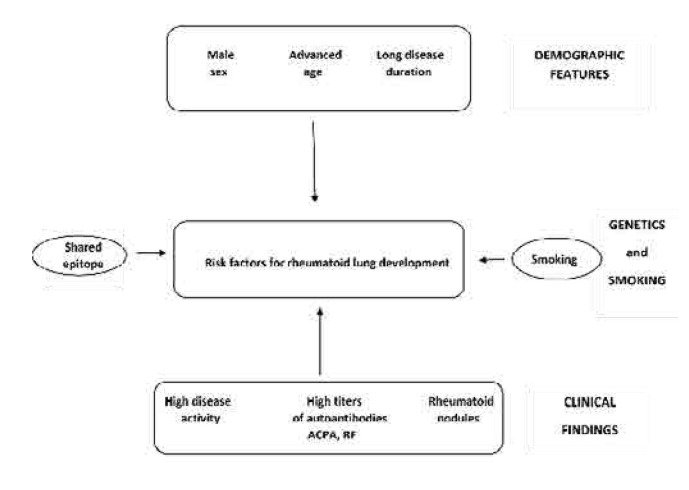
Demographic and clinical findings, along with genetic predisposition and lifestyle factors are responsible for rheumatoid lung development.

Recently, Wang et al. in a systematic review and meta-analysis of 56 studies reported a pooled prevalence of RA –ILD of 18.7%. The risk factors associated with ILD were male sex, older age, longer disease duration, smoking, the presence of autoantibodies, rheumatoid nodules, lung comorbidities, high RA disease activity and the use of steroids and leflunomide.^19^ Among these risk factors, smoking stands out as one of the strongest predictors of pulmonary involvement in RA, particularly in the development of airway disease and ILD. Smoking is also associated with the presence of RF, ACPA, greater disease severity, disease progression and may play a key role in the pathogenesis of RA.^30,31^ The risk factors for RA-associated lung disease are illustrated in **Figure 1**.

## RHEUMATOID LUNG DISEASE: CLINICAL AND DIAGNOSTIC CONSIDERATIONS

### RA airway disease

Studies have shown that 40%–60% of patients with RA may develop airway disease. Clinical features typically include an insidious onset of cough and productive sputum. Clinical examination reveals wheezing and decreased breath sound.^12,13^ PFT and HRCT are very useful while chest X-rays are usually normal. PFT show airflow obstruction and elevated residual volume. HRCT reveals bronchiectasis, bronchial wall thickening, bronchiolitis, air trapping and centrilobular opacities.^17,18^ Bronchiectasis in RA differs from other forms of bronchiectasis. Indeed, patients with RA and bronchiectasis exhibit more severe obstructive airway disease, increased susceptibility to recurrent lower respiratory tract infections and a faster decline in lung function. Moreover, they experience higher morbidity rate compared to individuals with bronchiectasis unrelated to RA.^32,33^

Airway diseases in RA remains relatively understudied and many patients are undiagnosed for extended periods. To address this, Matson et al. conducted a prospective, single-centre study assessing airway abnormalities in RA. In this single-centre study 188 patients with RA without clinical diagnosis of any respiratory disease, underwent HRCT scan and PFT. Airways obstruction was identified in 20,7% and was associated with older age, male sex and smoking. HRCT scan revealed airway abnormalities in 61% of the participants. More specifically, 55% had bronchial wall thickening, 12% had bronchiectasis and 5% had mosaic attenuation. These findings correlated with decreased forced expiratory volume (FEV_1_) and forced vital capacity (FVC), older age male sex and high RF. The authors concluded that airway abnormalities are frequently observed in RA patients. Thus, rheumatologists should be aware of this EAM of RA, as it often remains unrecognised in the majority of patients.^34^

### RA parenchymal disease

Parenchymal lung disease in RA known as RA-associated ILD, is one of the most prevalent and serious manifestations. It occurs in approximately 40–50% of RA patients. Chest X-rays detect ILD in about 5% of patients, HRCT in approximately 50% and lung biopsies or post-mortal studies reveal ILD in about 80% of patients. Clinical symptoms typically include an insidious onset of cough, exertional dyspnoea, fatigue and weakness. On physical examination, crackles or rales and decreased breath sound may be noted. Diagnosis involves a combination of imaging and diagnostic tests, including chest X-rays, HRCT, PFT, BAL and occasionally lung biopsy.^12,13^

**Figure 2.**
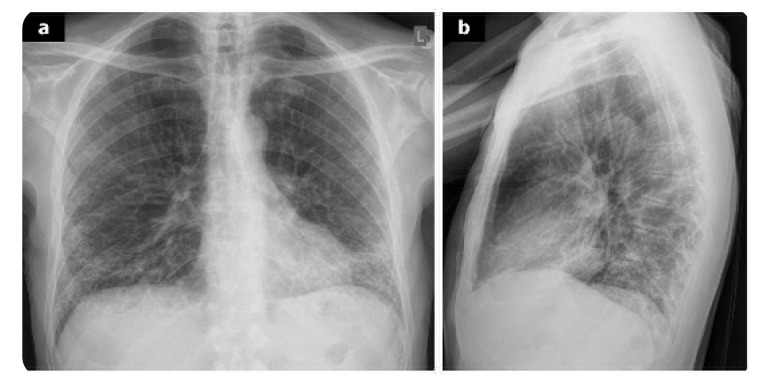
A 65-year old smoker with seropositive RA and dyspnoea on exertion. Antero-posterior (a) and lateral view (b) of x-rays show severe reticular opacities affecting the middle and lower zones of the lung.

**Figure 3 (left).**
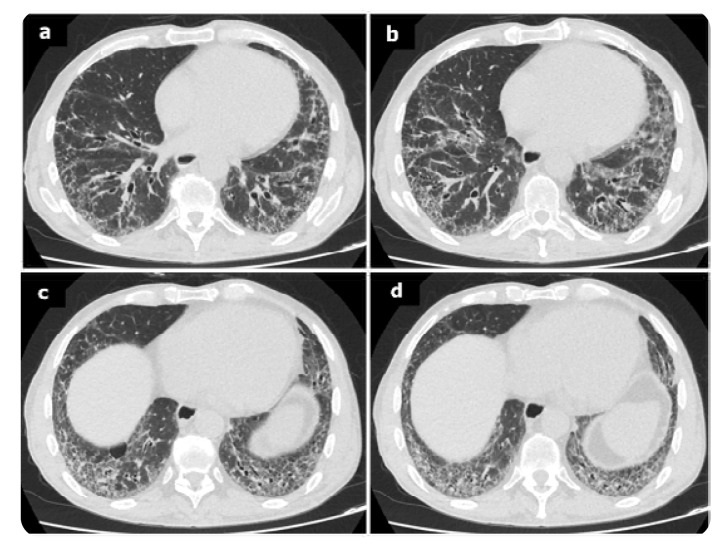
The same patient as in Figure 2. Axial HCRT scan of the chest with lung window settings reveal reticular opacities with traction bronchiectasis (**a,b,c**), without honeycombing and ground glass opacification mainly in the lower zones (**b,c,d**). A pattern of NSIP.

**Figure 4 (below).**
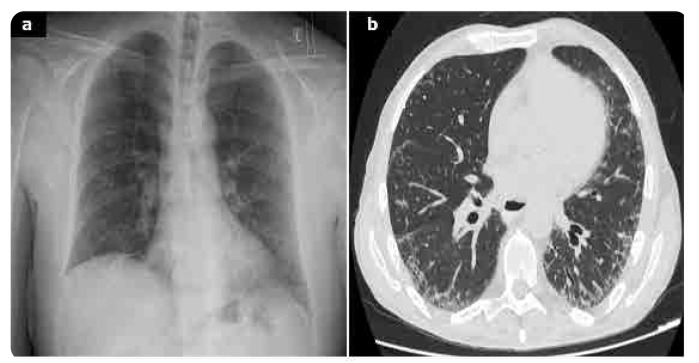
A 65year-old man, ex-smoker with seropositive RA with dry cough and dyspnoea on exertion. Antero-posterior x-rays of the chest reveals mild reticular opacities mainly in the lower zones with small amount of pleural effusions on the right side (**a**). Axial CT scan of the chest with lung window settings shows mild peripheral reticular opacities and traction bronchiectasis, without honeycombing compatible with a UIP pattern (**b**).

Chest X-ray is less sensitive in detecting ILD, typically revealing fibrotic reticulation especially at the lung basis in 5–7% of patients (**Figure 2**). In contrast, HRCT is considered to be the gold standard for ILD diagnosis. Different imaging patterns of HRCT in the setting of ILD have been reported. Usual interstitial pneumonia (UIP) is the most common and severe pattern in RA patients, followed by nonspecific interstitial pneumonia (NSIP). Other patterns described include organising pneumonia (OP), bronchiolitis obliterans, acute interstitial pneumonia and diffuse alveolar damage. Imaging findings characteristic of UIP comprise subpleural and basilar reticular abnormalities. Honeycombing with or without traction bronchiectasis may also be present (**Figure 3**).^14,15^ Histologically, UIP is characterised by areas of advanced fibrosis, fibroblastic foci, and microscopic honeycombing. In contrast, the NSIP pattern typically shows bilateral ground-glass opacities, with mild honeycombing abnormalities (**Figure 4**). In this setting histological findings comprise homogeneous cellular infiltrates with alveolar inflammation and interstitial fibrosis. PFTs are particularly useful, especially in asymptomatic patients. In RA-ILD, PFTs generally demonstrate a restrictive pattern and reduced diffusing capacity of carbon monoxide (DLCO). BAL findings in these patients are nonspecific. Lymphocytosis is the most common BAL abnormality in ILD, while neutrophilia is more characteristic of the UIP pattern. The prognosis of RA-ILD depends on the findings of HRCT patterns and the histological findings. Among the subtype, UIP is associated with the worst prognosis, similar to idiopathic interstitial pneumonia (IIP).^13–15^ Recently, Song et al. reported that infection-related deaths are the leading cause of mortality in this population.^35^ Moreover, high RA disease activity, low DLCO and the presence of the UIP pattern, are also independently associated with increased mortality in patients with RA-ILD.^36–38^

### Other parenchymal disease

Pulmonary rheumatoid nodules are also observed in patients with RA. First described by Caplan in 1953, these nodules can present either as single lesions or in clusters. They can appear before, during, or after the clinical onset of RA. Pulmonary rheumatoid nodules are more likely to develop in RA patients who have been exposed to inorganic dust, including asbestos, silica and coal.^13–15^ When a solitary nodule is present, it is essential to differentiate it from lung carcinoma. This requires evaluation using HRCT and lung biopsy (performed via ultrasound-guided or CT-guided techniques, bronchoscopy, or open biopsy) to establish a definitive diagnosis.^39^

### RA pleural disease

Pleural involvement in RA may present as pleurisy and/or pleural effusions. These effusions are typically small, asymmetric, and may wax and wane. Pleural disease is silent in some patients, while asymptomatic disease occurs in about 5% of RA patients. Large pleural effusions cause chest pain, dyspnoea, cough, and fever in some patients. On clinical examination, decreased breath sounds and a pleural friction rub may be detected. Chest X-ray is the initial imaging modality used, followed by HRCT for further evaluation.^12,13^ Thoracentesis with or without ultrasound guidance is useful in diagnosing pleural involvement and distinguishing it from other causes of pleurisy, such as infections and malignancies.

Among infectious diseases, viral infections are very common, followed by streptococcal and mycobacterial infections. Pleural fluid analysis plays a key role in differentiating these conditions. In RA, pleural fluid is typically exudate and characterised by low glucose levels (<50 mg/dl), low pH (<7, 3), elevated lactate dehydrogenase (LDH) (>1000 U/L) and also the presence of RF autoantibodies.^12– 15^ Microscopic examination of pleural fluid often reveals leucocytosis with mononuclear cells and macrophages. On the other hand, pleural fluid in infectious diseases typically shows marked leucocytosis with neutrophilia and cultures may be positive for the offending microorganism. For suspected mycobacterial infections, specific diagnostic tests should be performed.^13–19^

### RA vascular disease

Isolated pulmonary hypertension is very uncommon in RA. However, pulmonary hypertension may also occur in patients with ILD. Additionally, deep venous thrombosis (DVT) and pulmonary embolism (PE) have been reported in this population. Therefore, diagnostic tools such as ultrasound Doppler triplex, computed tomography angiography or magnetic resonance angiography should be used for its diagnosis.^12–15^ Capillaritis or alveolar haemorrhage may also occur in some patients and must be differentiated from infectious diseases using bronchoscopy, BAL along with HRCT.^12,13^

### Diagnostic screening

The 2023 ACR/American College of Chest Physicians (CHEST) recommendations for RA-ILD comprise screening tests for patients who present with signs or symptoms of ILD and have risk factors for ILD development. The screening tests include PFT (comprising spirometry, lung volume and diffusion capacity) as well as HRCT chest scan. Follow-up monitoring is advised every 3 to 12 months during the first year and less frequently once the disease is stable.^40^

Screening for RA-associated lung disease is crucial and rheumatologists play a key role in the early identification of affected patients, particularly those with RA-ILD.^15^ Based on published evidence, screening must be conducted in patients with respiratory symptoms such as cough and dyspnoea lasting for more than 3 months, in patients with evidence of clinical findings on lung examination, like crackles, rales, wheezing and decrease of breath sounds and finally, in patients without any clinical respiratory findings or signs who present several risk factors for lung disease development. These include: smokers, older male patients with the presence of high titres of autoantibodies (RF, ACPA) and high disease activity.^41^

The role of lung ultrasonography in screening for ILD in autoimmune rheumatic disease, including RA, has been investigated in recent years. The utility of lung ultrasonography in ILD has focused on evaluation of B-lines and the pleural line and diaphragm function. The B-lines are reverberation artifacts associated with septal thickening. They appear as hyperechoic vertical lines that originate at the pleural line and extend to the bottom of the screen, without fading. They move synchronically with lung expansion. Although B-lines can also be observed in other conditions such as pulmonary oedema and pulmonary haemorrhage and in conditions causing acute respiratory distress syndrome (ARDS), the presence of multiple B-lines indicates ILD.^15^

Lung ultrasonography has been predominantly used in patients with scleroderma-associated lung disease. Moreover, several studies have also evaluated the usefulness of lung ultrasonography in screening for ILD in RA patients. In a multinational cross-sectional study involving 203 RA patients, HRCT was used as the gold standard for ILD diagnosis. Among the participants, 26% had HRCT findings consistent with RA-ILD. Lung ultrasonography demonstrated a sensitivity of 83%, a specificity of 81,2% and a positive predictive value of 61,1%.^42^ In another cross-sectional study involving 106 RA patients, 32 (30,2%) were diagnosed with ILD based on HRCT. In this study, lung ultrasonography exhibited a sensitivity of 90,6% and negative predictor value of 94,7%.^43^ A recently published study confirmed these findings, further supporting the role of lung ultra-sonography as a promising tool for the early detection of RA-ILD.^44^ It serves as a complementary evaluation to clinical examination, helping to identify patients who are candidate for HRCT, which is the gold standard for confirming the diagnosis of ILD.^45^

## THE LUNG AS THE INITIAL SITE FOR RA PATHOGENESIS

RA pathogenesis is multifactorial, involving an interplay of genetic predisposition, environmental, hormonal and lifestyle factors. The interaction of these factors over time in individuals may give rise to the evolution, expression and outcome of RA.^46,47^ Among genetic factors, the Human Leucocyte Antigen (HLA) DR4 and the SE play a pivotal role in RA development. Several studies showed that the SE motif may be directly involved in disease pathogenesis by presenting the arthritogenic peptide, through antigen presenting cells (APC) to T-cells. The presence of SE also influences disease severity, including EAM and the generation of autoantibodies, RF and ACPA. Among environmental and lifestyle factors, infections, smoking, air pollution and occupational dust may contribute to RA phenotype expression.^37^ In fact, Klareskog et al. demonstrated a strong association between heavy smokers and ACPA development. The presence of SE was a risk factor only for ACPA positive RA patients. Furthermore, patients with double SE had 21-fold higher risk of developing ACPA positive RA, as compared to nonsmokers without SE.^30^

Citrullination is a post-translational modification of arginine to citrulline with new epitope formation and is attained by the presence of enzyme peptide-arginine deiminase (PAD) and high concentration of calcium.^48,49^ Proteins with the presence of residual citrulline such as vimentin, enolase, fibronectin and others have undergone citrullination. The conversion of arginine to citrulline enables the interaction between a high affinity citrullinated peptide and HLA-DRB1*-0401 MHC Class II molecules on APCs, which interact with T cells. This interaction contributes to B cell activation and promotes the production of ACPA.^50^ The question which arises here is where these immunological processes take place.

It is well established that RA-related autoantibodies (RF, ACPA) can be present in the serum 3 to 5 years, prior to joint inflammation. This period of autoimmunity, before the clinical onset of RA, is called “preclinical RA” or “at risk of RA”.^9,10^ Several studies have demonstrated that citrullinated peptides can be identified within the rheumatoid synovium membrane. However, citrullinated proteins have also been found in synovial membrane samples from patients with other inflammatory diseases and in those with osteoarthritis.^51^ Moreover, magnetic resonance imaging (MRI) and histological analysis of synovial biopsy from 13 individuals without arthritis, but positive for RF and/or ACPA antibodies, as well as from 6 healthy controls were evaluated prospectively. Over a mean follow-up period of 37 months, 4 out of 13 autoantibody-positive individuals developed arthritis, including one who met the revised ACR classification criteria for RA.^52^ Immunohistochemistry of synovial biopsies and MRI of the joints showed no differences between antibody-positive subjects and controls. These data suggest that the presence of citrullinated peptides in the inflamed synovium is not specific to RA, but rather may present an inflammation-associated phenomenon.^52^ Therefore, the high specificity of ACPA in RA cannot be explained by a unique expression of citrullinated peptides at the site of inflamed joints. It is more likely that RA patients exhibit an abnormal humoral response to citrullinated proteins, which may be present in any prior inflammation occurring outside the joints.^53^ Several investigators have demonstrated that the lung is a site of citrullination and this process is associated with smoking and chronic pulmonary diseases. Indeed, Klareskog et al. showed that smokers with pulmonary inflammation had a higher percentage of citrullinated cells in BAL fluid, compared to healthy nonsmokers and healthy smokers.^31^ Another study by Makrygiannakis et al. showed that smoking enhanced PAD2 expression in the bronchial mucosa and alveolar compartments, leading to the generation of citrullinated proteins. The study concluded that smoking is an environmental factor that may lead to citrullinated proteins breakup of tolerance in genetically susceptible individuals.^54^ As we mentioned previously, circulating autoantibodies were present prior to the onset of arthritis and this suggests that RA disease may initiate at any anatomical site apart from the joints. Indeed, circulating RF, especially IgA RF is present in “preclinical RA” period and has also been found in the sputum of these subjects. Considering that IgA RF antibodies are components of secretory mucosal immunological processes and the fact that IgA RF is also found in “preclinical RA”, it is possible to speculate that microbial alterations, or inflammation at mucosal level may represent the primary site of autoimmunity.^55,56^ These sites include mostly the lung, oral mucosa and intestinal mucosa. Periodontitis and antibodies against porphyromonas gingivalis are frequently observed in RA patients and the presence of these antibodies can also precede RA manifestations. Furthermore, gut microbial dysbiosis is also present in individuals at risk of developing RA.

**Figure 5.**
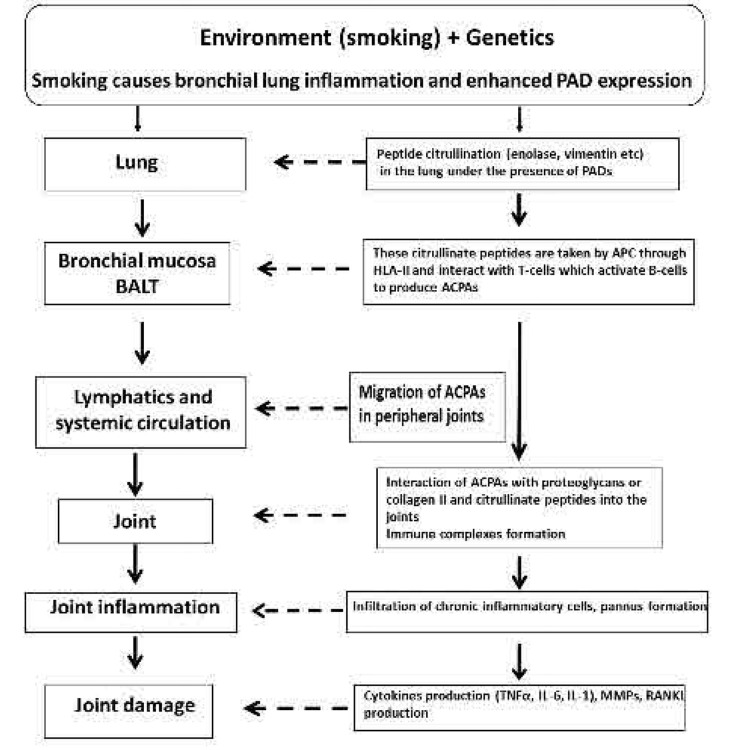
Schematic representation of RA pathogenesis.

Why in the lung? Certain studies suggest that the lungs may be the initial site of autoimmunity. Demoruelle et al. investigated the pulmonary abnormalities in 42 subjects who were positive for ACPA and/or RF but did not have inflammatory arthritis, as well as in 15 antibody-negative subjects and 12 patients with established early RA, using HRCT and PFT. HRCT scans revealed a high prevalence of airways abnormalities (70%), in autoantibody-positive subjects compared to 33% of antibody-negative controls. The prevalence and the type of lung abnormalities in antibody-positive subjects were similar to those observed in patients with early RA. These findings suggest that the lung may be an early site for the generation of RA-related autoimmunity.^57^ These data are supported by the fact that cigarette smoking causes chronic bronchial inflammation, enhances PAD2 expression and protein citrullination.^31,54^ It is of interest to note that the bronchial mucosa is rich in lymphoid tissue, known as “inducible bronchial associated lymphoid tissue” (iBALT), which correlates with tissue damage in the lung, where the immunological processes take place.^58^ More specifically, at the site of inflammation, activated neutrophils and mononuclear cells migrate through the endothelium and transmigrate into the bronchial mucosa, secreting proinflammatory cytokines and enhancing inflammation. In fact, neutrophils release PADs and undergo netosis, with the formation of neutrophil extra-cellular traps (NETs). NETs contain several citrullinated peptides, such as enolase, vimentin, and fibrinogen.^59^ These citrullinated peptides are processed by APCs, via the SE, interacting with T-cells and leading to B-cell activation and generation of ACPA autoantibodies.^55,56^ Identical citrullinated peptides are also present in the synovial membrane, which may serve as targets for immunological processes and disease perpetuation.^60^ In fact, through the lymphatics and systemic circulation, these autoantibodies migrate to peripheral joints, where they interact with proteoglycans or collagen II and joint citrullinated proteins leading to immune-complexes formation. These complexes attract immune cells, perpetuating inflammation by stimulating proinflammatory cytokines such as interleukin (IL)-1, IL-6 and tumour necrosis factor alpha (TNFα), causing joint damage and bone destruction (**Figure 5**). However, some important questions arise here. Why is immunological tolerance against the ACPA antigen lost? Is the immunological response attributed to a specific citrullinated protein, or do all citrullinated peptides have this capacity? Answering these questions is very difficult, as the mechanisms are not yet fully understood. In this direction, Verker et al. demonstrated a shared T-cell repertoire in pairs of BAL, blood and synovial samples from patients with early RA linked to smoking and SE. These data support a lung-joint axis in RA, suggesting that these T-cells and their T-cell receptors (TCRs) are selected and activated locally in the lung under the influence of smoking, eventually migrating to the synovial joints where they act as effector cells.^61^ However, further research is required to clarify these mechanisms.

## TREATMENT

In the last two decades, the introduction of biological therapies into the armamentarium for the management of inflammatory arthritides, has dramatically improved the clinical outcome of RA.^62,63^ However, the treatment of RA-associated lung disease, especially ILD, remains challenging, due to the lack of randomised controlled trials with robust data to guide clinical management. Several therapeutic agents have been suggested, including conventional synthetics disease modifying anti-rheumatic drugs (csDMARDs) and biological bDMARDs, along with immunosuppressive agents and Janus Kinase (JAK) inhibitors. Patients with RA and lung involvement, particularly those with RA-ILD have a higher risk and susceptibility to various infections, including viral, common and opportunistic infections. Since treatment with corticosteroids (CS) and bDMARDs can affect the immune response, this implies that rheumatologists should pay more attention to the interaction between treatment strategies, lung disease and infection risk. Therefore, before initiating any therapy, every RA patient should follow the vaccine program recommended by the international authorities and receive the recommended vaccines.^64^ In addition, screening for chronic or occult infections, such as hepatitis B and C viruses and mycobacterium tuberculosis is mandatory. Close follow-up with thorough physical examination at every visit and regular monitoring with appropriate blood tests and PFT, with imaging when indicated, is an imperative. Finally, smoking cessation should be strongly advised to all patients and consultation with a pulmonologist and pulmonary physiotherapist is mandatory. Pulmonary rehabilitation has been shown to improve exercise capacity breathing and improvement of quality of life.^65^ CS remain the mainstay of therapy for RA-ILD particularly in patients with NSIP or OP, where they can lead to radiographic regression and clinical improvement, but not in UIP patients. However, long term CS should be avoided.^66–68^ The recommended first-line agent for RA, MTX, has been associated with an increased risk of pneumonitis in some patients, which differs from RA-ILD because MTX pneumonitis is an acute or subacute hypersensitivity reaction which is treatable and reversible, in most cases.^65^ However, MTX has not been conclusively shown to induce or exacerbate underlying RA-ILD.^65,69^ Results from a multivariate analysis in early RA showed that MTX therapy was not associated with an increased incidence of RA-ILD. On the contrary, it seems that MTX may delay the onset of ILD.^70^ In patients with RA who developed respiratory symptoms while on MTX therapy, discontinuation and screening for ILD are advised.^71^ CS, in combination with azathioprine (AZA), have also been used for the treatment of RA-ILD. Despite its primary efficacy, recent studies have showed no effectiveness and increased adverse events in these patients.^65–69^ Cyclophosphamide (CP) is an immunosuppressive drug commonly used to treat patients with ILD, especially in scleroderma patients. However, there are no randomised trials to show its efficacy in RA-ILD.^68,69^ Mycophenolate mofetil (MMF), an immunosuppressive agent, has shown potential for stabilising or improving PFTs in ILD, (mainly scleroderma). While MMF may be effective in early or limited RA-ILD, evidence-based support is lacking and controlled studies are not yet available. MMF is also ineffective for articular manifestations of RA. Nevertheless, Saketkoo et al. reported clinical improvement in radiological stabilisation, along with improved PFT, in a small number of RA-ILD patients.^72^ A retrospective study also found a better survival rate of patients with RA-ILD who used MMF than those with AZA.^73^ In a series of 125 patients with autoimmune rheumatic diseases-ILD, including 18 RA patients, MMF was associated with mild improvement of FVC and DLCO.^74^ Overall, MMF combines reasonable efficacy with relatively low toxicity and may result in a modest improvement or stabilisation of PFT in RA-ILD.^74,75^

Anti-TNFα inhibitors have shown great efficacy in improving symptoms and radiographic progression in RA patients. Their use raised a concern for potential pulmonary toxicity in some patients.^62,63^ In contrast, some studies argue against any association between TNFα inhibitors and RA-ILD development.^68,69^ Rituximab (RTX) has shown promising results in ILD, especially in scleroderma patients.^76^ Small series of patients with RA-ILD showed some beneficial effects, however robust clinical data on the use of RTX in RA-ILD are missing^77–79^(**Table 2**). A retrospective analysis of RA-ILD patients treated with AZA (92 patients), MMF (77 patients) and RTX (43 patients) showed that immunosuppression led to improvement of PFT in a year.^80^

**Table 2.** Immunosuppressive drugs in the treatment of RA-ILD.

**Author**	**Study design**	**Number of patients**	**Study drug**	**Treatment duration**	**Results**
**Fui A.^77^**	Prospective	14	RTX	12 months	50% stable HRCT Patients with UIP worsened
**Md Yusof MY.^78^**	Observational	56	RTX	>10 years	52% stable FVC, DLCO 16% improved FVC, DLCO 12% worsened FVC, DLCO
**Vadillo C.^79^**	Longitudinal	68	RTX	>10 years	Fewer patients developed respiratory failure

**Monfredi A.^81^**	Retrospective	28	TCZ	30 months	56% stable FVC, DLCO 20% improved FVC, DLCO 24% worsened FVC, DLCO
**Otsuzi N.^82^**	Prospective	55	TCZ	6 months	Improvement of biological markers MMP-3, KL-6
**Suzuki K.^83^**	Retrospective	21	SAR	24 months	85,7% stable HRCT 2 cases improved 1 case worsened

**Tardella M.^84^**	Prospective	44	ABA	18 months	72,6% stable HRCT, PFT 16% improved HRCT, PFT 11,4% worsened HRCT, PFT
**Mena-Vázquez N.^85^**	Prospective Observational	57	ABA	27,3 months median	71,9% stable/improved HRCT 22,8% worsened HRCT 5,3% died
**López-Maraver M.^86^**	Retrospective Comparative 94iv vs 303sc	397	ABA	24 months	67% stable/improved HRCT (75%iv-64%sc)

RTX: Rituximab; TCZ: Tocilizumab; SAR: Sarilumab; ABA: Abatacept; HRCT: high resolution computed tomography; FVC: forced vital capacity; DLCO: diffuse lung carbon capacity; PFT: pulmonary function tests; IV: intravenously; SC: subcutaneously; MMP3: metalloproteinase3; KL-6: Krebs von der Lungen-6.

As regards the newer biological therapies, such as the T-cells costimulation blocker, abatacept (ABA) and tocilizumab (TCZ), or sarilumab (SAR) IL-6 receptor blockers, there are a few studies with a small number of patients and case series, showing some beneficial effects in RA-ILD patients^81–86^ (**Table 2**). The presence of ILD pattern did not significantly impact on immuno-suppression response. Indeed, Tardella et al. conducted a retrospective analysis of patients treated with JAK inhibitors or ABA, using HRCT scans after 18 months as an endpoint. Both therapies showed stabilisation or improvement in 83,9% and 88,6% respectively, with no significant differences between the two.^87^ Concerning the use of JAK inhibitors, in a study by Lee et al., 159 RA-ILD patients were matched with 477 RA-without ILD patients. The five years retention rate of biological and JAK inhibitors was lower in RA-ILD group compared to the control group. In the RA-ILD group, JAK inhibitors had the highest drug retention rate (64,3%), while the TNFα inhibitors had the lowest retention rate (30,6%). Approximately 58,5% of RA-ILD patients and 48,4% with RA-without ILD discontinued treatment, mainly due to adverse events or lack of efficacy. Logistic regression analysis showed that current smoking negatively affected drug retention, while CS had a protective effect against withdrawal.^75^ A systematic review and meta-analysis, reported that JAK inhibitors were well tolerated among 183 with RA-ILD patients. The pooled analysis showed that JAK inhibitors were well tolerated and could be a viable treatment option for RA-ILD patients, offering safety and efficacy comparable to ABA and RTX.^88^ It seems that JAK inhibitors have shown some promising results of stabilising the PFT in RA-ILD.^87,88^ Anti-fibrotic agents may be used in severe cases.^89^ A systematic review by Cassone et al. reported that ABA showed an improvement in PFT by 16.6%, stabilisation by 74.9% and worsening by 8.5%. On the other hand, TCZ exhibited an improvement of 17%, stabilisation by 65.5% and worsening by 17%. Finally, RTX showed an improvement of 5.4%, stabilisation 76.6% and worsening 16.9%.^67^ The 2023 ACR/CHEST guidelines recommend MMF, or AZA, or RTX with small doses of CS as first-line therapy for RA-ILD. Additional options include the use of CP. For patients with progressive disease, the addition of anti-fibrotic agents is suggested.^90^

To summarise the above studies, there is now evidence that MTX has more protective than detrimental effects in RA-ILD development. The use of CS, even in high doses, is recommended for patients with NSIP or OP, but not in UIP, and long-term use should be avoided. Furthermore, the current evidence suggests the avoidance or cessation of TNFα inhibitors in patients with a new diagnosis of ILD. In these patients RTX or ABA are recommended.^65,66^ Finally, TCZ or JAK inhibitors may be considered as a second-line biological therapy.^87,88^ Anti-fibrotic agents, such as nintedanib or pirfenidone, may be added to these therapies and could have synergistic protective effects.^89,90^ There is a significant heterogeneity in the treatment of patients with RA-ILD. Those with UIP pattern have a worse prognosis and clinical outcome, compared to patients with NSIP pattern. However, even NSIP patients may show disease progression over time, including lung function decline and death. Recently, Brooks et al. reported that patients with RA-ILD represent a high-risk population for development of lung cancer.^91^ Enhanced screening may therefore be warranted to achieve early diagnosis and reduce mortality in these patients.

The lack of robust evidence in RA-ILD treatment complicates treatment decision-making. Given the high risk of morbidity and mortality associated with RA-ILD, regardless of radiological patterns, there is an urgent need for randomised controlled trials to guide therapies in this population.

## CONCLUSIONS

Rheumatoid lung disease is a common EAM in RA patients, most commonly presenting as airway disease, ILD and pleural manifestations. Several risk factors are associated with lung disease development, including smoking, older age, male sex, high disease activity and high titres of autoantibodies. The pathogenesis of RA is still unknown, involving genetic and environmental factors. Among the environmental factors, smoking is very crucial since it causes chronic bronchial inflammation, enhances PAD2 expression, protein citrullination and the generation of autoantibodies. It seems that the lung may be an early site of the generation of RA autoimmunity. Morbidity and mortality in RA with lung disease are very high and the treatment is challenging. Early recognition of lung involvement is essential. Close follow-up and monitoring, along with the appropriate pharmacological treatment are mandatory, taking into account that immunosuppressive drugs can alter immune response leading to the development of infections. Thus, the management of RA-related lung disease should be based on a multidisciplinary approach, involving collaboration with pulmonologists and individualised treatment based on the needs of the patient. Non-pharmacological therapies may benefit patients with RA-ILD. Pulmonary rehabilitation has showed to improve quality of life. Vaccinations against influenza, streptococcal infection, herpes zoster, SARSCov2 and advice on smoking cessation counselling should be provided. Despite the progress made with the use of biological and JAK inhibitors, there are still no prospective randomised trials, specifically focused on RA-ILD patients. Several immunosuppressive drugs, including MMF, RTX, ABA, TCZ and JAK inhibitors, are used in this setting, but robust evidence supporting their efficacy is still missing. Anti-fibrotic agents such as nintedanib and pirfenidone may provide an additional protective effect. However, further research in this topic is needed.

## STATEMENT OF ETHICS AND CONSENT

All presented material is published after written consent of the patients, although sensitive data and personal details are not included in the publication.

## CONFLICT OF INTEREST

The authors declare no conflicts of interest.

## AUTHOR CONTRIBUTIONS

All authors contributed equally to the study as well as to the preparation of the manuscript for publication. A.A. Drosos: Conceptualisation, manuscript preparation, final review, A.K. Zikou: Imaging evaluation, editing, M. Yerolatsite: Literature screening, editing, P.V. Voulgari: Review, editing. All authors have approved the final submitted version and all authors have the responsibility for the integrity and accuracy of the work.

## FUNDING

None.
